# Data for the physical and mechanical properties of high volume fly ash cement paste composites

**DOI:** 10.1016/j.dib.2017.11.047

**Published:** 2017-11-16

**Authors:** Ertug Aydin, Hasan Şahan Arel

**Affiliations:** a Department of Civil Engineering, European University of Lefke, Lefke, Mersin 10, North Cyprus, Turkey; b İzmir University, Gürsel Aksel Bulvarı, No. 14, 35350, Üçkuyular, İzmir, Turkey

## Abstract

The data presented herein are compiled of the research summary of “Characterization of High-Volume Fly-Ash Cement Paste for Sustainable Construction Application” (Aydin and Arel, 2017) [1]. This data article provides general information about the ASTM Class C and Class F fly ash cement paste composites composed of silica fume, lime, water reducing admixtures in three different level of workability (0 mm, 100 mm and 200 mm). The dataset here also helps the readers to understand the links with the basic properties of the ingredients, for example, how can porosity be predicted based on the mixture design? how can the strength of the material be linked with the basic strengths of the constituent ingredients?.

**Specifications Table**

**Table:** 

Subject area	*Civil Engineering, Material Science Engineering*
More specific subject area	*Mix design parameters*
Type of data	*Images, Figures, Text File*
How data was acquired	*Physical and mechanical tests (Laboratory)*
Data format	*Raw, Analyzed*
Experimental factors	*The seven different mixture groups, three slump range (0 mm, 100 mm and 200 mm), three testing age (7, 28 and 90 days) high volume fly ash, cement, lime, silica fume and water reducers are used to manufacture the cement paste composites in a cylindrical mold.*
Experimental features	*Various amount of high volume fly ash are replaced with cement to investigates the effects of mixture ingredients on mixture design in controlled low strength applications.*
Data source location	*Mersin 10 Turkey, Lefke, North Cyprus*
Data accessibility	*The all data herein are available within this article.*
Related research article	E. Aydin and H.Ş. Arel “Characterization of High-Volume Fly-Ash Cement Paste for Sustainable Construction Applications”, Const. Build. Mater. 2017, (underreview].

**Value of the data**

• The data presented herein can be used to investigate the effects of high volume fly ash on mixture proportions
• The dataset can be used by others to construct mix design monograph for cement pastes
• The data presented herein may be used to develop new methods for controlled low strength applications
• The data may be concerned with the effect of different fly ash content in pore volume of cement paste.
• The research data may be helpful for manufacturing commercially sustainable building products

## 1 Data

**Fig. 1:**
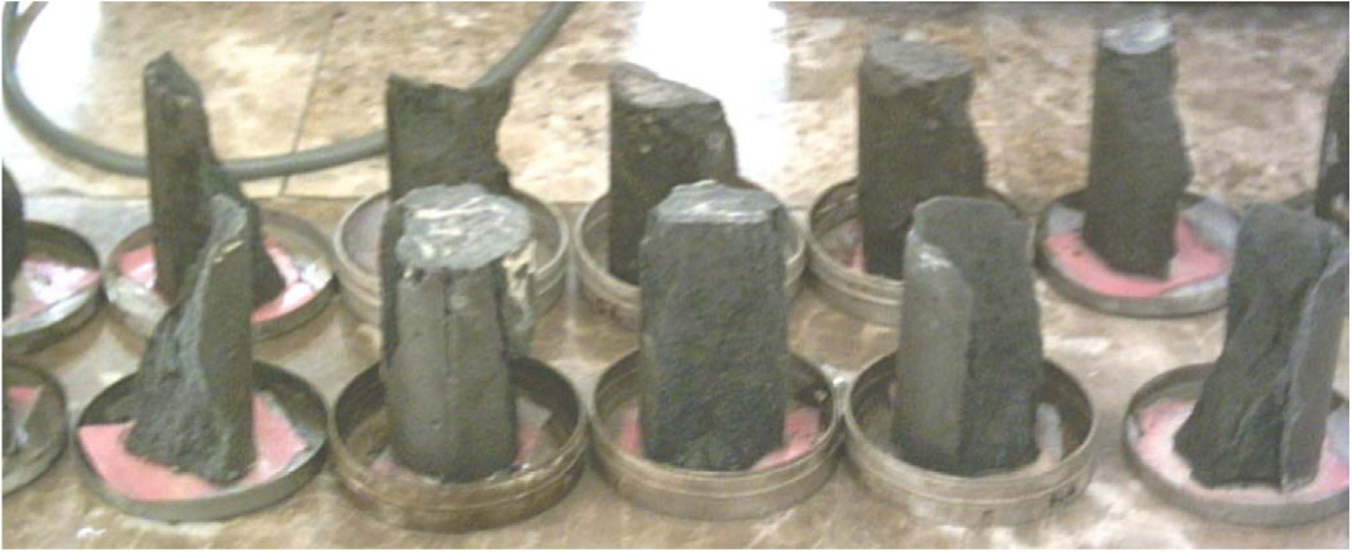
Selected fracture samples.

**Fig. 2:**
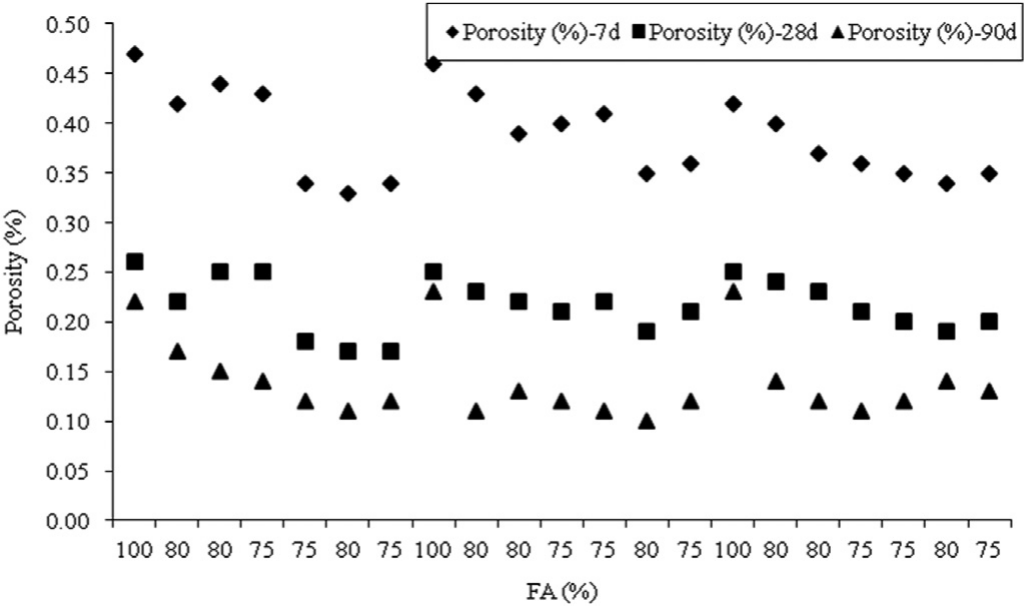
Effects of ASTM Class C Fly ash on porosity at 7, 28 and 90-days.

**Fig. 3:**
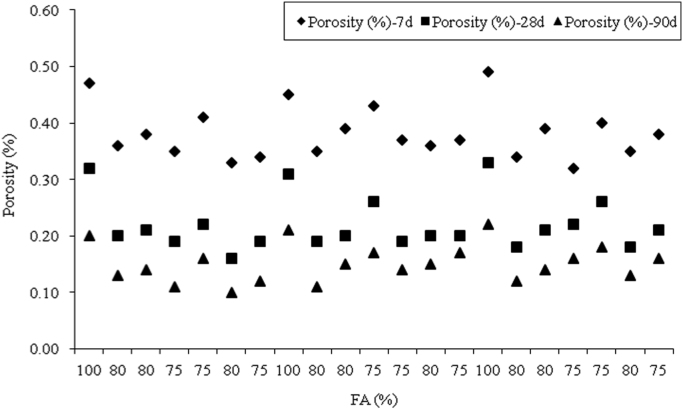
Effects of ASTM Class F Fly ash on porosity at 7, 28 and 90-days.

**Fig. 4:**
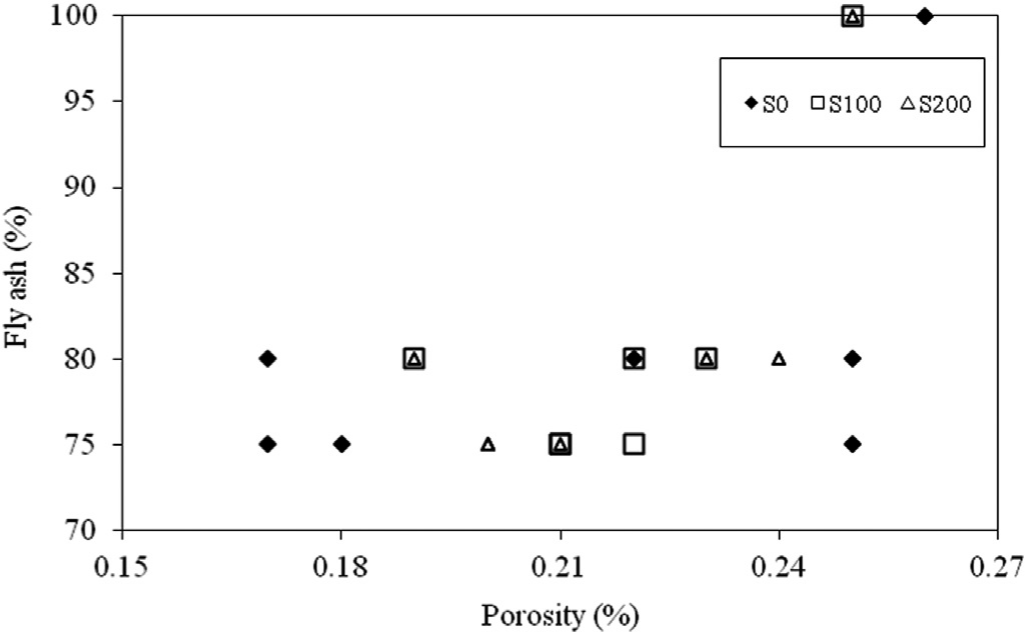
Effects of ASTM Class C Fly ash on porosity for 0 mm, 100 mm and 200 mm slump value.

**Fig. 5:**
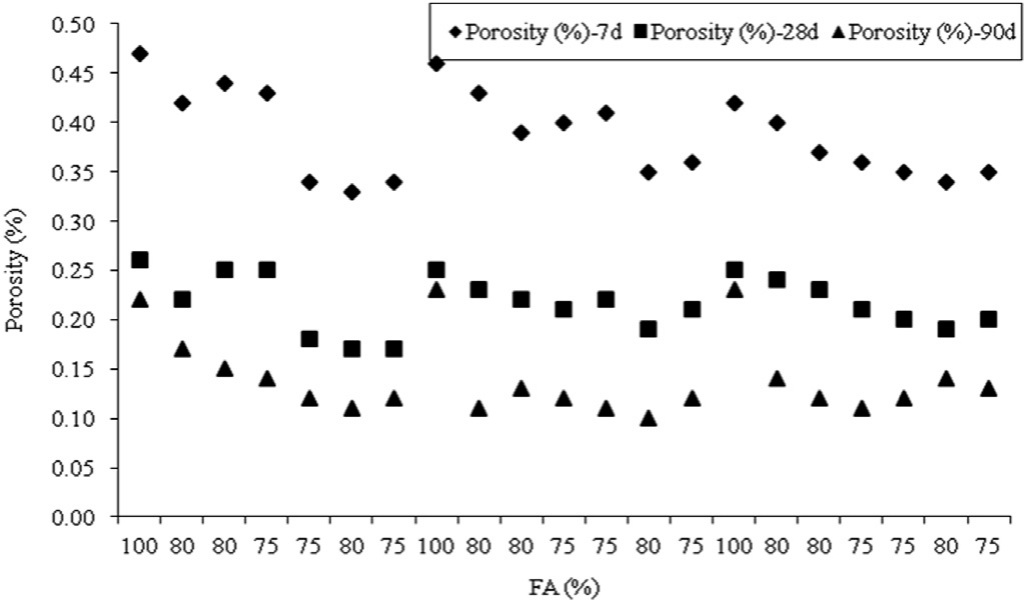
Effects of ASTM Class F Fly ash on porosity for 0 mm, 100 mm and 200 mm slump value.

**Fig. 6:**
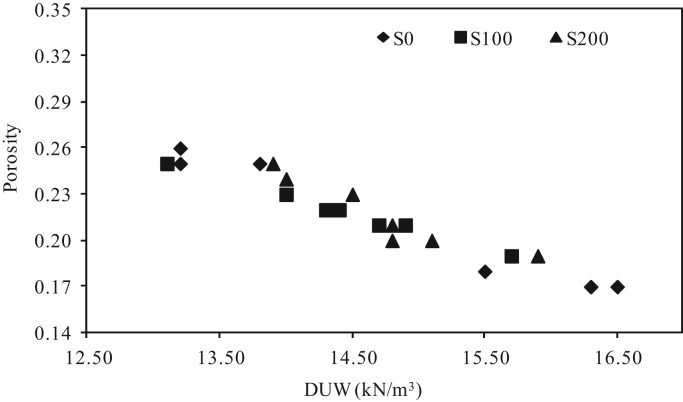
Effects of porosity on dry unit weight for 0 mm, 100 mm and 200 mm slump value for ASTM Class C fly ash cement paste composites at 28-days.

**Fig. 7:**
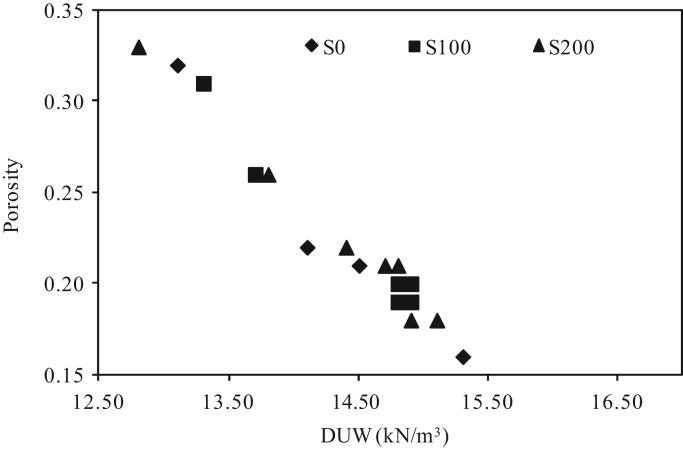
Effects of porosity on dry unit weight for 0 mm, 100 mm and 200 mm slump value for ASTM Class F fly ash cement paste composites at 28 days.

**Fig. 8:**
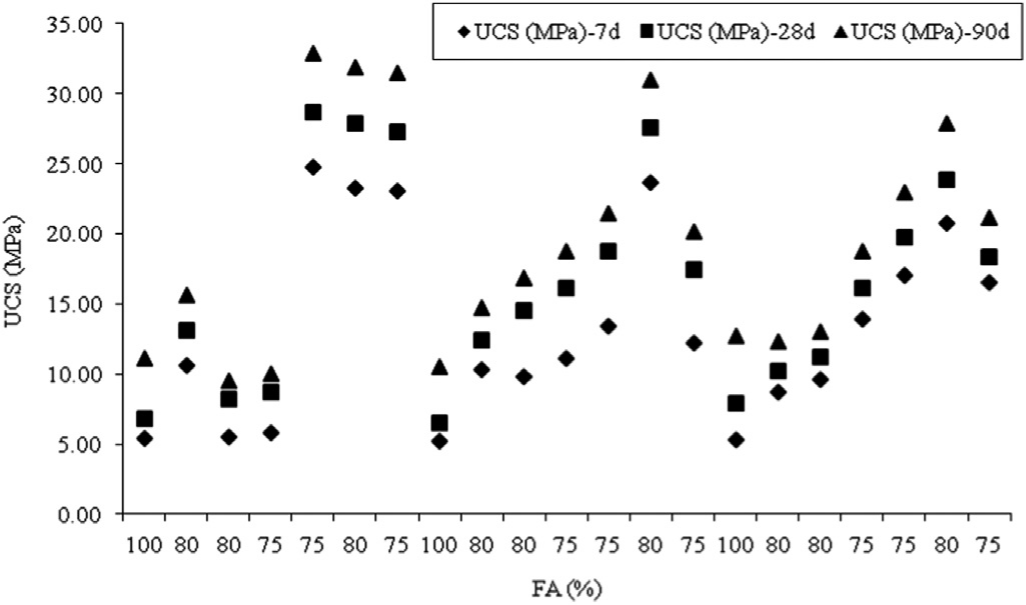
Effects of fly ash on UCS for 0 mm, 100 mm and 200 mm slump value for ASTM Class C fly ash cement paste composites at 7, 28 and 90 days.

**Fig. 9:**
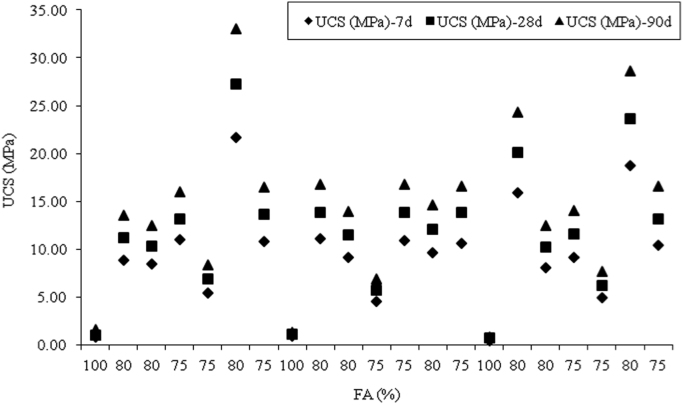
Effects of fly ash on UCS for 0 mm, 100 mm and 200 mm slump value for ASTM Class F fly ash cement paste composites at 7, 28 and 90 days.

The dataset presented herein were obtained from the physical and mechanical tests of various amounts of ASTM Class C and Class F fly ash, silica fume, lime and water reducers to produce high volume fly ash (HVFA) cement paste composites. The data provides in this article composed of pure cement paste composites. The detailed of the dataset presented here can be found in [1]. Additionally, the existing models proposed in previous studies for mortars and cement paste [2], [3], [4], [5], [6], [7] were used to check the applicability in controlled low strength applications. The regression analysis of test data for 1512 samples were used to predict the effects of mixture ingredients in those applications to exhibit novel mixture design methods (Fig. 1, Fig. 2, Fig. 3, Fig. 4, Fig. 5, Fig. 6, Fig. 7, Fig. 8, Fig. 9).

## 2 Experimental design, materials and methods

The water-to-binder (w/b) ratio was adjusted to attain the required workability. This article investigates three workability range (0 mm, 100 mm and 200 mm) and seven mixture groups. The groups are all optimized in a previous researches [2], [3], [4], [5]. The data presented here examined the mixture ingredients of fly ash, cement, silica fume, lime and water reducers. The seven hundred fifty six samples for each type of fly ash class are used. Various HVFA cement paste mixes were experimentally examined and 1512 mixes are evaluated. Composites were cast in 55 mm by 110 mm cylindrical molds and vibrated through vibrating table. The produced samples were tested in 7, 28 and 90 days. The detailed of mix proportions, experimental setup and results can be found in [1].

## References

[1] Aydin E., Arel H.Ş. (2017). Characterization of high-volume fly-ash cement paste for sustainable construction applications. Const. Build. Mater..

[2] Aydin E. (2016). Novel coal bottom ash waste composites for sustainable construction. Constr. Build. Mater..

[3] Aydin E., Balkis A.P. (2017). Preliminary study on the durability properties of high-volume fly ash mortar composites. ASTM J. Test. Eval..

[4] Aydin E. (2006). Utilization of High Volume Fly Ash Cement Paste in the Manufacture of Building Materials (PhD thesis).

[5] E. Aydin, Utilization of high volume fly ash cement paste in civil engineering construction sites, in: Proceedings of the Fifth International Conference on Construction in the 21st Century (CITC-V), Collaboration and Integration in Engineering, Management and Technology, May 20-22, Istanbul, Turkey. pp. 1526–1535, 2009.

[6] Dolado J.S., van Breugel K. (2011). Recent advances in modeling for cementitious materials. Cem. Concr. Res..

[7] Aydin E. (2017). Staple wire-reinforced high-volume fly-ash cement paste composites. Constr. Build. Mater..

